# Monogenic interferonopathy presenting as CMV infection in infancy

**DOI:** 10.1186/1546-0096-13-S1-P12

**Published:** 2015-09-28

**Authors:** C Schütz, C Frisch, M Hoenig, J Crow, K Schwarz, K Debatin, A Schulz

**Affiliations:** 1 University Medical Center Ulm, Department of Pediatrics, Ulm, Germany; 2 Université Paris Descartes, Hôpital Necker-Enfants Malades, Laboratory of Neurogenetics and Neuroinflammation, Institut Imagine, Paris, France; 3 Ulm University, Institute for Clinical Transfusion Medicine and Immunogenetics, Ulm, Germany

## 

The patient is the 4th child of a consanguineous Turkish couple. She was diagnosed with CMV- pneumonitis at 7 months of age. In addition she presented with pernio-like skin lesions of cheeks and ear lobes. Immunologically she was hypergammaglobulinemic for her age (IgG 11,5 g/l), her T-cell subpopulations, T-cell-proliferation to mitogens, number of B- and NK-cells were normal. IL2 and INFg production was diminished upon stimulation. Histology of lung tissue revealed alveolitis with infiltration by macrophages and histiocytes as well as lymphofollicular hyperplasia with activation of germinal centres.

At the age of 8 y/o the patient was diagnosed with pulmonary hypertension. She is being treated with sildenafil since the age of 12 y/o in addition to continuous oxygen. At the age of 15 years the patient presented for the first time with a vasculitic rash of arms, upper legs and feet. Immunofluorescence showed a positive lupus band. Immunologically she is persistently hypergammaglobulinemic (IgG 18,4 g/l) and has an upregulated interferon signature.

Although the presumed diagnosis in infancy was a functional T-cell deficiency, the disease course points towards a lupus-like disease with chronic pneumonitis and skin vasculitis as well as development of an antibody-profile compatible with SLE. Known genes for monogenic SLE including TMEM173 were excluded. Results of whole exome sequencing are pending.

